# Anisotropy-free arrayed waveguide gratings on X-cut thin film lithium niobate platform of in-plane anisotropy

**DOI:** 10.1038/s41377-024-01506-1

**Published:** 2024-06-29

**Authors:** Junjie Yi, Changjian Guo, Ziliang Ruan, Gengxin Chen, Haiqiang Wei, Liwang Lu, Shengqi Gong, Xiaofu Pan, Xiaowan Shen, Xiaowei Guan, Daoxin Dai, Kangping Zhong, Liu Liu

**Affiliations:** 1 State Key Laboratory of Extreme Photonics and Instrumentation, College of Optical Science and Engineering, International Research Center for Advanced Photonics, Zhejiang University, Hangzhou, 310058 China; 2 Guangdong Provincial Key Laboratory of Optical Information Materials and Technology, South China Academy of Advanced Optoelectronics, South China Normal University, Higher-Education Mega-Center, Guangzhou, 510006 China; 3 National Center for International Research on Green Optoelectronics, South China Normal University, Guangzhou, 510006 China; 4 Photonics Research Institute, Department of Electrical and Electronic Engineering, The Hong Kong Polytechnic University, Hung Hom, Kowloon, Hong Kong (SAR) China; 5 Jiaxing Key Laboratory of Photonic Sensing & Intelligent Imaging, Intelligent Optics & Photonics Research Center, Jiaxing Research Institute Zhejiang University, Jiaxing, 314000 China

**Keywords:** Optical materials and structures, Integrated optics

## Abstract

Arrayed waveguide grating is a versatile and scalable integrated light dispersion device, which has been widely adopted in various applications, including, optical communications and optical sensing. Recently, thin-film lithium niobate emerges as a promising photonic integration platform, due to its ability of shrinking largely the size of typical lithium niobate based optical devices. This would also enable multifunctional photonic integrated chips on a single lithium niobate substrate. However, due to the intrinsic anisotropy of the material, to build an arrayed waveguide grating on X-cut thin-film lithium niobate has never been successful. Here, a universal strategy to design anisotropy-free dispersive components on a uniaxial in-plane anisotropic photonic integration platform is introduced for the first time. This leads to the first implementation of arrayed waveguide gratings on X-cut thin-film lithium niobate with various configurations and high-performances. The best insertion loss of 2.4 dB and crosstalk of −24.1 dB is obtained for the fabricated arrayed waveguide grating devices. Applications of such arrayed waveguide gratings as a wavelength router and in a wavelength-division multiplexed optical transmission system are also demonstrated.

## Abstract (web summary)

A universal strategy to realize anisotropy-free dispersive components, such as arrayed waveguide gratings, on a uniaxial in-plane anisotropic thin-film lithium niobate platform is introduced for the first time.

## Graphical abstract

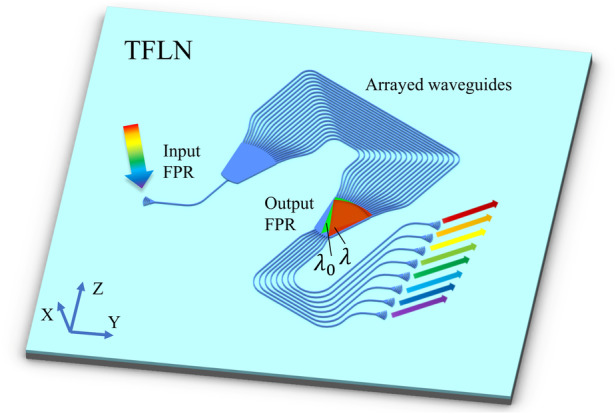

## Introduction

Light dispersion device is one key and historic component in optics, which can be used to split light with different wavelengths into different directions. Bulk dispersion components, such as prisms and gratings, has been widely used in nowadays optical systems for a wide range of applications^1^. The integrated solution for such a light dispersion component is more attractive due to its compact size, easy integration with other optical devices, and scalability. Among different solutions, arrayed waveguide grating (AWG) is one of the most successful and versatile one, which uses an array of waveguides with different lengths to control the dispersion of the device^2^. This renders it very convenient to design its performance figures including channel spacing, channel number, filter bandwidth, etc., to fit different applications. A stand-alone AWG device can already be used as a wavelength (de)multiplexer or router in high-capacity optical communication systems, or as a spectrometer in optical sensing and imaging applications^2–6^. By integrating AWGs with other photonic components including semiconductor amplifiers, modulators/detectors, or switches, multi-functional integrated photonic circuits have also been demonstrated, such as, multi-wavelength lasers^7^, reconfigurable optical add-drop multiplexer^8,9^, microwave photonic processers^9,10^, or optical computing chips^11^. High-performance AWGs have been demonstrated in various photonic integration platforms based on silica^12,13^, polymer^14^, InP^15^, silicon^16–19^, or silicon nitride^20,21^ in the last several decades.

Recently, thin-film lithium niobate (TFLN) emerges as a promising photonic integration platform, which has drawn attentions in both industry and academia^22,23^. Due to the high electro-optic (EO) coefficient and fast EO response of lithium niobate material, combined with the high index contrast waveguide structure, the TFLN platform has enabled EO modulators with an ultra-high bandwidth, a low insertion loss, and a low drive voltage^24–28^. Besides, TFLN based nonlinear photonic devices and acousto-optic devices have been demonstrated with performances surpassing other platforms^29–31^. Through heterogeneous integration, lasers and detectors have also been realized on TFLN with a high integration density^32–37^. Considering these developments in technologies, it would be very interesting if a scalable dispersion component, e.g., an AWG, can be built on TFLN. This would initiate lots of applications based on a single TFLN chip. Unfortunately, it is not as intuitive as one might think to design an AWG on TFLN, since lithium niobate is a uniaxial anisotropic material. It exhibits ordinary refractive indices in two axes (the X and Y axes), and extraordinary refractive index in the other axis (the Z axis). Furthermore, to fully exploit the predominant EO coefficient, an X-cut wafer structure, where the extraordinary optical axis lies within the wafer plane, and the extraordinary polarization, where the dominant electrical field also lies within the wafer plane, are mostly adopted^24–28^. In this case, the light would see different refractive indices, when propagating in different directions on a wafer. This anisotropic thin film has never been delt with before in other high-index contrast platforms. It has been shown that this in-plane anisotropy on X-cut TFLN brings difficulties in designing even a simple straight waveguide or a waveguide bend^38^. Naturally, one may think that it would be very difficult, or if not impossible, to design an AWG in this case, since the phase and group delays of the arrayed waveguides in an AWG have to be controlled accurately with respect to each other. The existing AWGs demonstrated recently on TFLN still show poor performances^39–42^, even when they were mostly based on the Z-cut film, where the wafer surface remains isotropic.

In this paper, we report, for the first time, high-performance AWGs based on the X-cut TFLN platform. A universal strategy to design anisotropy-free dispersive components on an in-plane anisotropic photonic integration platform is introduced, which can be employed for not only AWGs in this study but also other dispersion components. Various configurations of AWGs for wavelength (de)multiplexers at C-band with 400 GHz and 200 GHz channel spacings, as well as an 8×8 wavelength router, are realized on X-cut TFLN. The fabricated devices exhibit the best insertion loss of 2.4 dB and crosstalk of −24.1 dB. Such a device is successfully adopted in a wavelength division multiplexing (WDM) optical transmission system.

## Results

### Anisotropy-free design concept

**Fig. 1:**
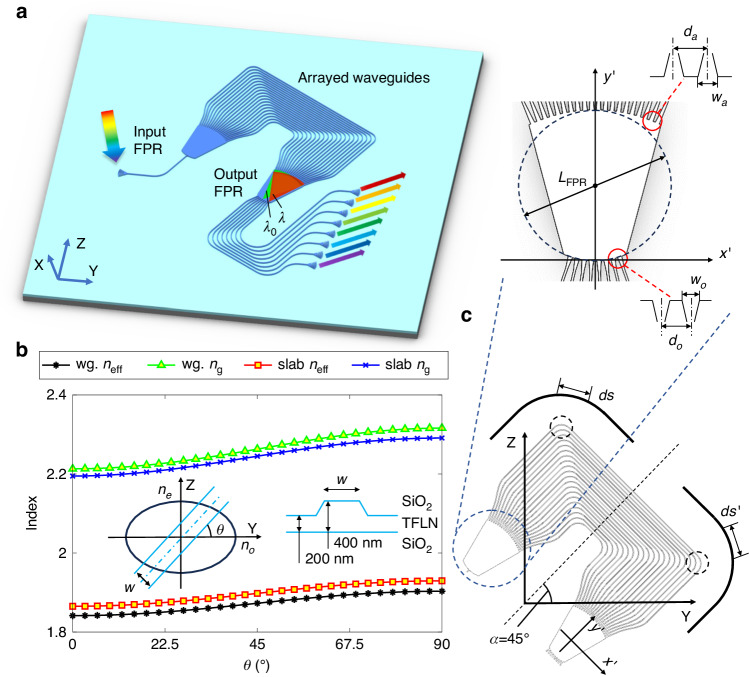
**a** Sketch of an AWG and its working principle. **b** Effective and group indices of the TE0 mode in a ridge waveguide and a slab waveguide on X-cut TFLN with different propagation directions *θ*. The inset shows the model and structure of the waveguide. Here, the original slab thickness of the waveguides is 400 nm. The width *w* and etching depth of the ridge waveguide is 2 μm and 200 nm, respectively. **c** Structure and parameters of the proposed AWG design with its symmetrical axis *α* along 45°

The basic structure of an AWG, as plot in Fig. 1a, consists of two free propagation regions (FPRs) and an array of waveguides with different lengths. The entrance/exit pupils of the FPRs to the arrayed waveguides are arranged in a circular line to avoid spherical aberration, i.e., to ensure a proper focusing. The key element in this AWG structure is the waveguide array, which should bring a designed dispersion for the light entering the output FPR, and the wavelength splitting function is therefore realized. Generally speaking, two conditions should be fulfilled to ensure the above processes to work.

I. At the central wavelength (marked as ${\lambda }_{0}$), each waveguide in the array should have the same phase, so that at the entrance pupil of the output FPR a wave-front follows the circular pupil edge is generated. Then, light at ${\lambda }_{0}$ will focus on the central output waveguide. The phase delay ${{{\varnothing }}}_{j}$ of a waveguide at ${\lambda }_{0}$ can be expressed as:

1 $${{{\varnothing }}}_{j}=\frac{2\pi }{{\lambda }_{0}}{\int }_{L}{n}_{{\rm{eff}}}\left(\theta \right){ds}$$

where, ${n}_{{\rm{eff}}}$ is the effective index of the waveguide mode at ${\lambda }_{0}$, *j* = 1,2…*N* is an integer which indicates the waveguide number, and *N* is the total number of waveguides in the array. The integral here follows the route *L* of each arrayed waveguide, and *ds* is the arc differential along the waveguide. Since an in-plane anisotropic film is discussed here, as shown in Fig. 1b, ${n}_{{\rm{eff}}}$ of the fundamental transverse electrical (TE0) mode is a function of the propagation direction *θ* of the waveguide on the wafer plane, which is also the local tangent of the waveguide route *L*. As discussed above, the phase generated by each waveguide, i.e., ${{{\varnothing }}}_{j}$ wrapped within 2π, should be the same for all *j*. This is referred as the phase delay condition below.

II. At an arbitrary non-central wavelength $\lambda$, the arrayed waveguides should create a dispersion (or phase difference) which is proportional to differences in the wavelength $\Delta \lambda$ and the waveguide number, so that a tilted circular wave-front is generated at the entrance pupil of the output FPR. Then, light at $\lambda$ will focus at a different output position. We can express this dispersion ${\Delta {{\varnothing }}}_{j}$ at each waveguide using the first order approximation as:

2 $$\Delta {{{\varnothing }}}_{j}={{{{\varnothing }}}_{j}|}_{\lambda }-{{{{\varnothing }}}_{j}|}_{{\lambda }_{0}}=\frac{2\pi \Delta \lambda }{{\lambda }_{0}^{2}}{\int }_{L}{n}_{g}\left(\theta \right){ds}$$

where, ${n}_{g}$ is the group index of the waveguide mode at ${\lambda }_{0}$, which is also a function of *θ*. As discussed above, $\Delta {{{\varnothing }}}_{j+1}-\Delta {{{\varnothing }}}_{j}$, should be the same for all *j*. This is referred as the group delay condition below.

In an in-plane isotropic platform, such as silicon, silica, or Z-cut TFLN, the integrals in Eqs. (1) and (2) are simply reduced to the length *L*_*j*_ of each arrayed waveguide, since ${n}_{{\rm{eff}}}$ and ${n}_{g}$ are now irrelevant to *θ*. Then, the above two conditions can be easily met when the length difference of adjacent arrayed waveguides is the same, i.e., ${L}_{j+1}-{L}_{j}=m{\lambda }_{0}$, where *m* is noted as the diffraction order of the AWG. This trivial AWG design has been widely adopted for almost all its previous demonstrations.

However, on an in-plane anisotropic platform, such as X-cut TFLN as shown in Fig. 1b, ${n}_{{\rm{eff}}}$ and ${n}_{g}$ of a typical waveguide structure exhibits a large variation at different *θ*. The trivial AWG design with a fix waveguide length difference is no longer applicable here (see Supplementary Note 2). Due to the circular pupil of the FPRs, the path of each arrayed waveguide follows a non-Manhattan route. It is then difficult to find simple expressions for Eqs. (1) and (2) in general cases. The aforementioned phase and group delay conditions become also nontrivial. One solution is to fine-tune the path or pattern of each arrayed waveguide to fulfill these conditions. However, this is a brute-force approach that requires a lot of design efforts and most likely with a tight fabrication tolerance. We will also show, in Supplementary Note 2, that it could be generally hard to achieve both delay conditions simultaneously in this way. A smarter and robust procedure is needed to cope with this difficulty.

From Fig. 1b, one can realize that the variation trends of ${n}_{{\rm{eff}}}$ and ${n}_{g}$ are likely to follow a trigonometric relation, which can be fitted as:

3 $${n}_{{\rm{eff}}(g)}(\theta )={n}_{{\rm{eff}}(g)}(0{^{\circ}} ){\cos }^{2}\theta +{n}_{{\rm{eff}}(g)}(90{^{\circ}}){\sin }^{2}\theta$$

We will show, in Supplementary Note 1, that Eq. (3) is actually a theoretical approximation for the refractive index of optical waves in uniaxial anisotropic material, and ${n}_{{\rm{eff}}(g)}\left(\theta \right)$ from rigorous numerical computations fits very well to this equation. We can then think of a special AWG layout which has a symmetrical axis direction *α* along 45°, as shown in Fig. 1c. In this case, for any *ds* of the arrayed waveguide in a direction of *θ*, there would exists a symmetrical counterpart *ds’* in a direction of 90°—*θ*. Therefore, Eqs. (1) and (2) can be rewritten as:

4 $${{{\varnothing }}}_{j}=\frac{2\pi }{{\lambda }_{0}}\frac{\left[{n}_{{\rm{eff}}}\left(0{^{\circ}} \right)+{n}_{{\rm{eff}}}\left(90{^{\circ}} \right)\right]}{2}{\int }_{L}{ds}=\frac{2\pi }{{\lambda }_{0}}{n}_{{\rm{avg}},{\rm{eff}}(g)}{L}_{j}$$

5 $$\Delta {{{\varnothing }}}_{j}=\frac{2\pi \Delta \lambda }{{\lambda }_{0}^{2}}\frac{\left[{n}_{g}\left(0{^{\circ}} \right)+{n}_{g}\left(90{^{\circ}} \right)\right]}{2}{\int }_{L}{ds}=\frac{2\pi \Delta \lambda }{{\lambda }_{0}^{2}}{n}_{{\rm{avg}},{\rm{eff}}(g)}{L}_{j}$$

where ${n}_{{\rm{avg}},{\rm{eff}}(g)}=\frac{\left[{n}_{{\rm{eff}}(g)}\left(0{^{\circ}} \right)+{n}_{{\rm{eff}}(g)}\left(90{^{\circ}} \right)\right]}{2}$ is the average effective (group) index of the waveguide mode along 0° and 90°. One can find that the $\theta$ dependence, i.e., the anisotropy, is removed in Eqs. (4) and (5). The conventional AWG design can be adopted here by only taking ${n}_{{\rm{avg}},{\rm{eff}}(g)}$ as the waveguide mode indices and putting the AWG layout in a 45° symmetrical fashion. An anisotropy-free AWG design is then achieved in this special case on the X-cut anisotropic TFLN platform.

### Device structure

**Fig. 2:**
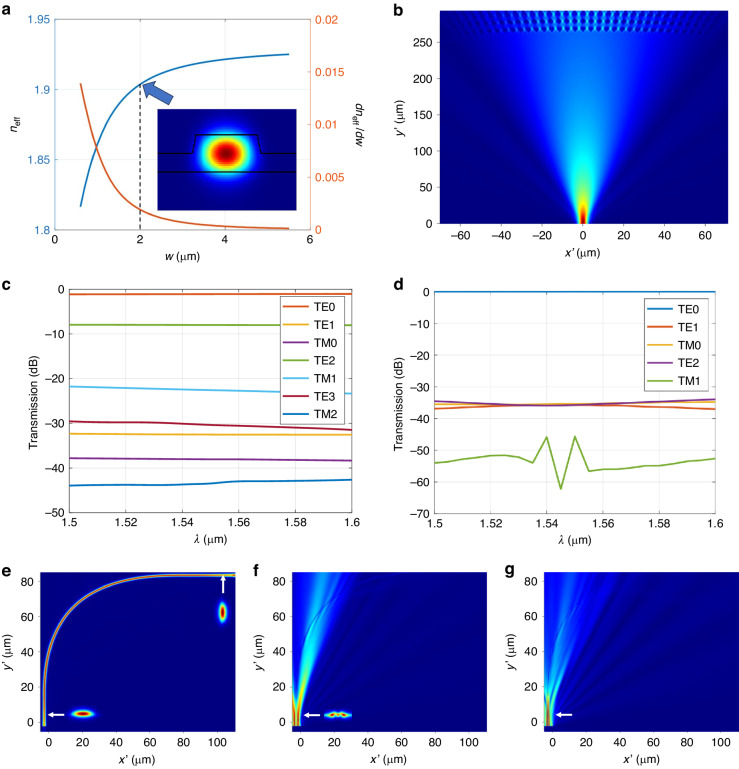
**a** Effective indices and their change rate with respect to the width of the TE0 mode in the TFLN ridge waveguide at different widths *w*. **b** Light propagation in the input FPR. **c** Total transmissions to different modes in all the arrayed waveguides from the input FPR. **d** Transmissions from the TE0 mode to other modes in the 90° compact bend in the arrayed waveguide. **e**–**g** Light propagation in the bend for TE0, TE2, and TM1 modes, respectively. Here, Design-3 from Table 1 is adopted

Based on the above strategy, we designed four AWGs of different configurations centered at ${\lambda }_{0}$ = 1550 nm, and their key structural parameters are listed in Table 1. When designing an AWG, an appropriate diffraction order *m* is selected firstly to ensure that all channels can fit in one FSR. Then the structure parameters of the arrayed waveguides at the pupils of the FPRs, including the arrayed waveguide number *N*, the arrayed waveguide width ${w}_{a}$, and the arrayed waveguide spacing ${d}_{a}$, are optimized taking into account the insertion loss, as well as the resolution and scale of fabrication processes. These parameters are then used to determine the length of the FPR ${L}_{{\rm{FPR}}}$ and the input/output waveguide width ${w}_{o}$ considering the light diffraction in the FPR. Finally, the input/output waveguide spacing ${d}_{o}$ is calculated using the designed channel spacing. Due to the large size, it is challenging to simulate the entire AWG in one numerical simulation domain. A three-stage strategy is therefore adopted, including, a finite-difference time-domain simulation of the input FPR, a semi-analytical formula for the arrayed waveguides, and a two-dimensional scalar diffraction model for the output FPR. Besides their routes, the cross-sectional structure of the arrayed waveguides is also one important part of the AWG device. Here, a shallowly-etched ridge waveguide structure is employed as shown in Fig. 1b. The waveguide width is chosen large, i.e., 2 μm, which is well in the multimode region. In this case, as compared to the single mode region, the effective index of TE0 mode is not sensitive to the changes in width, as shown in Fig. 2a. This approach has been proved effective for reducing the random phase error resulted from fabrication variations for phase sensitive devices ^19^.

**Table 1:** Parameters of designed AWGs at C-band

	Configuration	*m*	*N*	${w}_{a}$ (μm)	${d}_{a}$ (μm)	${L}_{{\rm{FPR}}}$ (μm)	${w}_{o}$ (μm)	${d}_{o}$ (μm)
Design-1	1 × 4 × 400 GHz	80	16	2.9	3.4	120	3.3	3.9
Design-2	1 × 8 × 400 GHz	46	32	2.0	2.4	100	2.5	3.9
Design-3	1 × 8 × 200 GHz	80	32	2.9	3.4	260	4.2	7.7
Design-4	8 × 8 × 200 GHz	99	32	2.0	2.4	100	2.5	4.4

Figure 2b shows the light propagation in the input FPR. Here, parameters of Design-3 from Table 1 are considered. Clearly, a mismatch between the slab mode and the waveguide mode is present, which leads to the excitation of higher-order modes in the arrayed waveguides. We analyzed the total excited power in each waveguide mode of all the arrayed waveguides, and results are shown in Fig. 2c. One can find that although the fundamental mode (TE0) power remains high, in a broad wavelength range of 1550–1600 nm and with a loss less than 1 dB, there is still a substantial amount of power coupled to higher-order modes. Specifically, the excited TE2 and TM1 mode powers are the largest two, about −8 dB and −22 dB, respectively. Since multimode waveguides are adopted in the array, these higher-order modes can possibly propagate along them, and would result in undesired crosstalk at the output of the AWG device.

To eliminate this impact, we implement compact 90° bends with a narrower width (0.7 μm) in the arrayed waveguides except the straight sections as shown in Fig. 1c. These bends, which consists of a pair of 45° modified Euler-bends and a pair of 25-μm-long adiabatic tapers, also serve as filters for higher-order modes. To minimize mode mismatch between the bent and straight sections, we selected a maximal radius of 300 μm for the Euler-bends. Simultaneously, a minimal radius of 50 μm was chosen to ensure a low transmission loss for the fundamental mode and a compact footprint. This design results in an effective radius of 82 μm for the waveguide bends. As illustrated in Fig. 2d, e, the TE0 mode propagates smoothly through the bend with only a negligible loss. On the contrary, Fig. 2f, g demonstrate that the TE2 and TM1 modes are effectively blocked with both transmissions less than −45 dB.

**Fig. 3:**
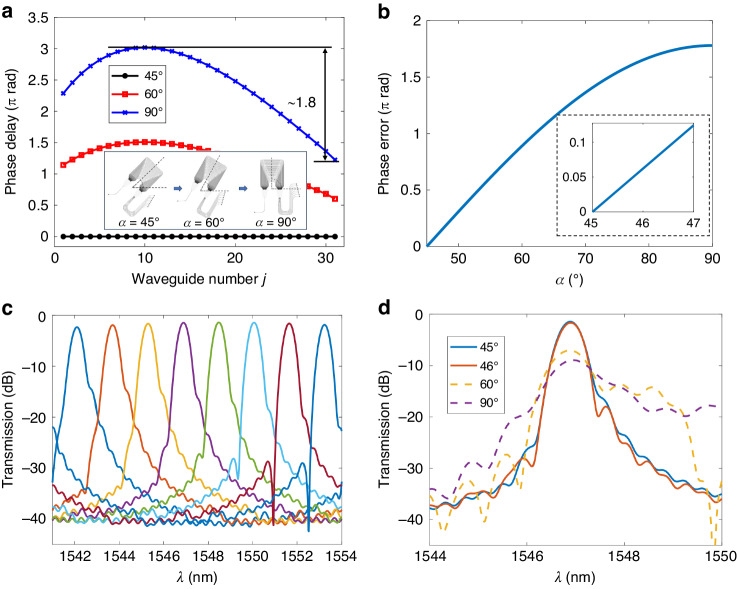
**a** Simulated phase delays ${{{\varnothing }}}_{j}$ in each array waveguide when the symmetrical axis *α* of the AWG is along 45°, 60°, and 90°. ${{{\varnothing }}}_{j}$ is relative to the case of *α* = 45°. **b** Phase errors generated in the arrayed waveguides at different *α*. The inset shows the region near *α* = 45°. **c** Simulated output spectra at *α* = 45°. **d** Simulated output spectra of one channel with different *α*. Here, Design-3 from Table 1 is adopted

According to the previous derivation, achieving an anisotropy-free AWG is possible on X-cut TFLN when it is symmetrically placed along the 45° axis of the material. However, maintaining this angle exactly during fabrication can be an issue. We further conducted an analysis for the induced phases from the arrayed waveguides when the direction of the symmetrical axis *α* of an AWG deviates from 45°. Here, the AWG design is again based on Design-3 in Table 1. Clearly, as shown in Fig. 3a, the phase delay ${{{\varnothing }}}_{j}$ from each arrayed waveguide is no longer the same when *α* ≠ 45°, where the phase delay condition would not be met. We mark the largest difference between the phase delays from the arrayed waveguides as the phase error. As shown in Fig. 3b, this phase error exhibits a monotonical increasing with respect to *α*. Under the ideal condition, i.e., *a* = 45°, the spectral response exhibits a low insertion loss and crosstalk as shown in Fig. 3c. We also simulated the spectral responses of one channel with different *α* as shown in Fig. 3d. This angle deviation directly causes a waveform distortion, resulting in a significant degradation in performance. Normally, ±1° angular alignment accuracy can be guaranteed in either the cut of the lithium niobate crystal itself or the current lithography tools. In this limit, the phase error is maintained also small within ±0.05 π, which only brings negligible impacts on performances, according to Fig. 3b, d. We can then conclude that the proposed design strategy can indeed support a robust AWG implementation on X-cut TFLN.

### Fabrication and measurement

We fabricated the designed AWGs in Table 1 on a commercial lithium-niobate-on-insulator wafer with 400 nm thick X-cut top lithium niobate layer. The full fabrication processes include electron-beam lithography (EBL), dry etching, and over-cladding deposition (see Materials and methods). As mentioned before, a ridge waveguide structure, where 200 nm of lithium niobate was etched, was used here. This structure has been mostly adopted for making, e.g., modulators^27,28^. Besides AWGs, grating couplers were also prepared at each end of input and output waveguides for light in- and out-couplings during measurements.

**Fig. 4:**
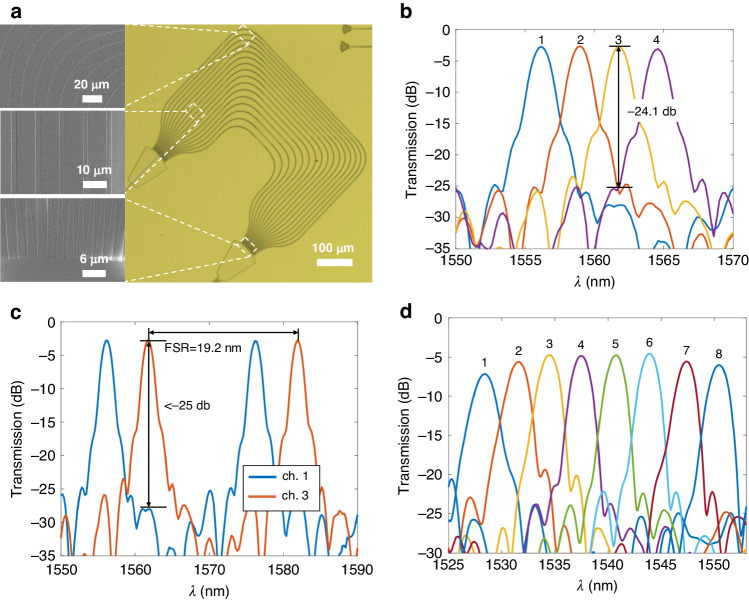
**a** Pictures of one fabricated AWG. **b**, **c** Output spectra of the AWG using Design-1 from Table 1. The measured channel spacing is 391 GHz. **d** Output spectra of the AWG using Design-2 from Table 1. The measured channel spacing is 416 GHz. The numbers on the wavelength peaks mark the corresponding output channels

First, Design-1 in Table 1 with 400 GHz channel spacing was fabricated and measured. Figure 4a presents some pictures of the AWG and some key parts of it, showing the good quality of this finished device. The footprint of this AWG is about 0.8 mm × 0.9 mm. Figure 4b, c show the measured transmission spectra at the 4 output ports. Here, the measured insertion loss for the central channel is about 2.4 dB, and a non-uniformity of about 1.2 dB to the edge channels. Additionally, the crosstalk between adjacent channels is about −24.1 dB. Between non-adjacent channels, the crosstalk is lower than −25 dB. To the best of our knowledge, this is so far the best for AWGs on TFLN. The free spectral range (FSR) of this AWG was measured at 19.2 nm, which is matched well to the simulation result. A similar design was expanded to an 8-channel AWG of 400 GHz channel spacing, i.e., Design-2, and the results are shown in Fig. 4d. Similar performances were obtained for this design, except slightly degraded insertion loss and crosstalk. This is most likely due to the increased number of arrayed waveguides, where the random phase error resulted from fabrication variations becomes more severe.

**Fig. 5:**
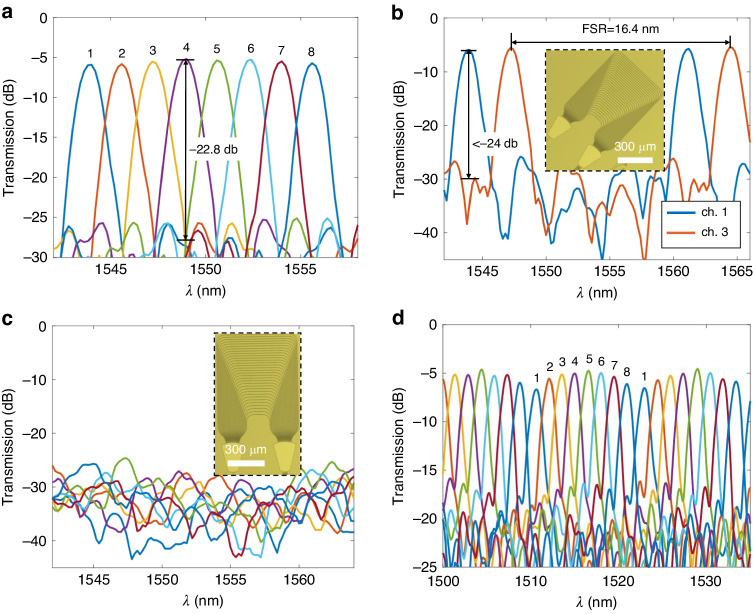
**a**, **b** Output spectra of the AWG using Design-3 from Table 1. The measured channel spacing is 207 GHz. **c** Output spectra of an AWG using the same parameters as those in (**a**) and (**b**) except *α* = 90°. **d** Output spectra of an AWG router, i.e., Design-4 from Table 1, from one input channel to all output channels. The measured channel spacing is 209 GHz. The numbers on the wavelength peaks mark the corresponding output channels

To further demonstrate the scalability of the present design approach, a larger AWG of 200 GHz channel spacing was also implemented, i.e., Design-3 in Table 1. Pictures and measured responses of this device are shown in Fig. 5a, b. The footprint of this design is about 1.7 mm × 1.8 mm. The measured insertion losses are 4.8 dB for central channels and 5.7 dB for edge channels. The crosstalk between adjacent and non-adjacent channels are −22.8 dB and <−24 dB, respectively. The FSR of this device is 16.4 nm, also matched well to the simulation. As a comparison, we also implemented the same AWG design, while putting its symmetry axis on the Z axis of the material, i.e., *α* = 90°. As shown in Fig. 5c, the measured output spectra do not show any meaningful filtering or multiplexing responses. This experimentally proves the effectiveness of the present strategy to remove the anisotropy of an AWG in this case.

Besides wavelength (de)multiplexing functions, we also implemented an AWG based 8 × 8 wavelength router^9^, where the FSR of the device is designed equal to the channel spacing multiplied by the channel number. Therefore, the eight wavelength channels in each input waveguide are distributed among all eight output waveguides, respectively. The parameters of this device are listed as Design-4 in Table 1. Figure 5d shows the measured wavelength responses from one input channel to the 8 output channels, showing the desired cyclic wavelength responses. The full responses of such a device are shown in Supplementary Note 3.

Table 2 summarizes some key metrics of AWGs demonstrated on the TFLN platform in recent years. Apparently, the present devices mark the first AWGs realized experimentally on X-cut TFLN. Their insertion loss and crosstalk performances even overpass those of AWGs on Z-cut TFLN, which is not bothered by difficulties of on-chip anisotropy.

**Table 2:** Comparison of several performance metrics for AWGs on TFLN

Crystal cut	Insertion loss (dB)	Crosstalk (dB)	Configuration	Ref.
Z-cut	25	−15	8 × 8 × 500 GHz	^39^
Z-cut	3.32	−3.82	1 × 8 × 200 GHz	^40^
Z-cut	6.6 8.4	−19.3 −18.3	1 × 8 × 1.25 THz 1 × 16 × 500 GHz	^41^
Z-cut	13	−6	1 × 100 × 6.25 GHz	^42^
X-cut	2.4 3.9	−24.1 −20.6	1 × 4 × 400 GHz 1 × 8 × 400 GHz	This work
X-cut	4.8 5.1	−22.8 −12.7	1 × 8 × 200 GHz 8 × 8 × 200 GHz	This work

### High-speed data transmission

**Fig. 6:**
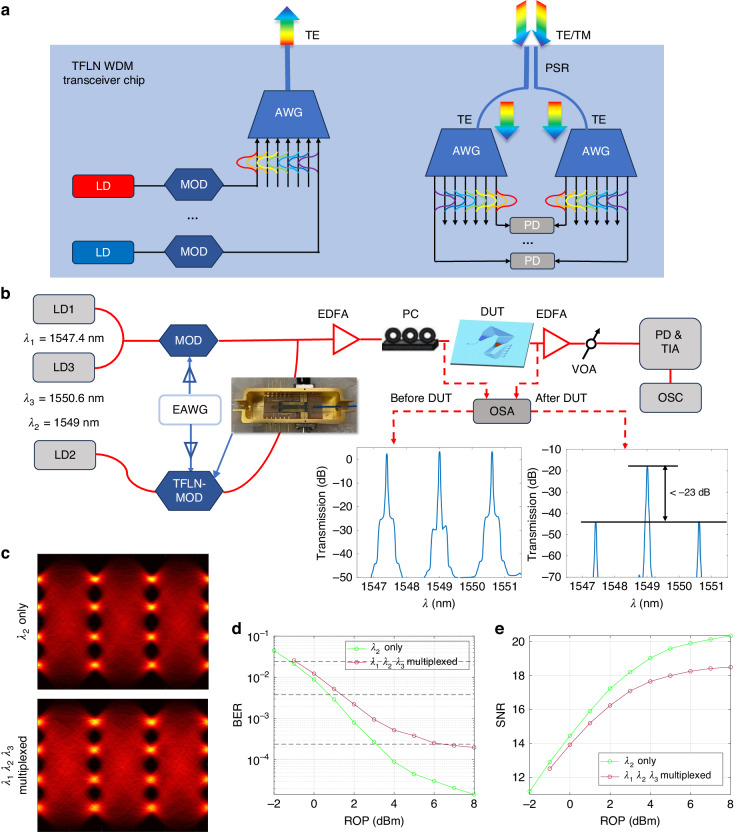
**a** Sketch of a TFLN based WDM transceiver chip with heterogeneous integrated lasers and detectors. Other components are all on TFLN. **b** Measurement setup for WDM data transmissions using the present AWG for wavelength demultiplexing and a packaged TFLN modulator. **c** Measured 56 GBaud PAM-4 eye-diagrams after equalization for the central channel ${\lambda }_{2}$ with ${\lambda }_{1}$ and ${\lambda }_{3}$ activated and deactivated at 8 dBm ROP. **d**, **e** Measured BERs and SNRs at different ROPs for the channel ${\lambda }_{2}$ with ${\lambda }_{1}$ and ${\lambda }_{3}$ activated or deactivated. LD laser diode, PD photo-diode, TIA transimpedance amplifier, MOD modulator, PC polarization controller, EDFA erbium-doped fiber amplifier, VOA variable optical attenuator, OSA optical spectrum analyzer, OSC oscilloscope, EAWG electronic arbitrary wave generator, TM transverse magnetic, PSR polarization splitter and rotator, DUT device under test

The implementation of AWGs on the X-cut TFLN platform facilitates integrating WDM functionalities with high-performance EO devices. Figure 6a shows a WDM based optical interconnect system. With the present AWG designs, all of the building blocks in this system, except the lasers and detectors (which in principle can also be integrated using heterogeneous approaches), can be realized monolithically on a single TFLN chip. To test the present AWGs used as a wavelength demultiplexer, a practical multiwavelength transmission system is built (see Materials and methods) as shown in Fig. 6b. Here, three wavelengths were employed, which are aligned to the three adjacent channels of the AWG in Fig. 5a. A 56 GBaud four-level pulse amplitude modulated (PAM-4) data signal was put on the central wavelength channel using a lab-made and packaged TFLN modulator as shown in the inset of Fig. 6b, which is fabricated using the same technology and platform as those of the present AWG. The data with the same format and baud rate on the two adjacent side channels was created using a commercial lithium niobate modulator. All three wavelengths were sent to the input waveguide of the AWG, and the output from the central wavelength channel was monitored. This setup helps analyze the influence of the crosstalk to the transmitted data. Figure 6c–e show the performances of the received optical signal at the central channel when the two side channels were activated or deactivated. One can find that good quality eye-diagrams can be obtained in both cases. The measured bit-error-rates (BERs) at different received optical powers (ROPs) indicate power penalties of about 0.8 dB for 3.8 × 10^−3^ BER and about 3 dB for 2.4 × 10^−4^ BER considering crosstalk from the adjacent channels in the present AWG demultiplexer.

## Discussion

In conclusion, a universal strategy for designing AWGs on a photonic integration platform of a uniaxial in-plane anisotropy, e.g., X-cut TFLN, has been introduced. By aligning the symmetrical axis of an AWG along the 45° axis of the lithium niobate material, an anisotropy-free design can be achieved. This renders AWG in this case compatible to conventional designs in an isotropic platform. Following this strategy, we have successfully demonstrated high-performance AWGs on the X-cut TFLN platform at C-band. To the best of our knowledges, this is the first AWG device of this kind experimentally. The fabricated AWGs of 400 GHz channel spacing exhibit an insertion loss of 2.4 dB and a crosstalk of −24.1 dB between adjacent channels. For 200 GHz channel spacing AWGs, an insertion loss of 4.8 dB and a crosstalk of −22.8 dB has been obtained. We have also implemented an 8 × 8 AWG based wavelength router, and evaluated the high-speed data transmission performances of the present AWG used as a wavelength demultiplexer. The proposed design strategy can be readily adopted for realizing other AWG configurations with more channels and more arrays. As an example, an 100 channel AWG spectrometer with 6.25 GHz channel spacing is designed and analyzed (see Supplementary Note 2).

The successful implementation of AWGs on X-cut TFLN facilitates integrating this scalable wavelength filters with high-speed EO tuning and modulating devices on a single TFLN chip. This enables not only WDM transceiver chips mentioned in this paper, but also other high-performance, multi-functional, and reconfigurable photonic circuits. It is also worthwhile to note that the anisotropy-free design strategy here is based solely on the basic waveguide characteristics, and is not confined only for AWGs and TFLN. In fact, any other devices, such as lattice filters based on Mach-Zehnder interferometers^43^, that require certain defined phase and group delay relations, or any other in-plane uniaxial anisotropic platforms, such as thin-film lithium tantalate^44^, can also adopt this universal strategy to remove the anisotropy in their designs. The measured AWG performances here are still limited by the phase errors resulted from the fabrication variation in the widths of the arrayed waveguides. After all, the fabrication technologies for TFLN devices are still less matured that those for, e.g., silicon based photonic circuits. For the present devices, this variation most likely came from the proximity effect of the EBL process employed to define the arrayed waveguide patterns. This problem could be relieved by using, e.g., stepper lithography in a wafer scale.

## Materials and methods

### Sample fabrication

The fabrication of the AWG devices started from a commercial lithium-niobate-on-insulator wafer (Novel Si Integration Technology), consisting of 400 nm thick top X-cut TFLN layer, 3 μm thick buried oxide layer, and 500 μm silicon substrate. 100-keV EBL (Vistec 5200) was used to pattern a negative resist layer (ma-N 2403) spun on top of the wafer. The pattern was transferred directly from the resist to the TFLN layer using inductively-coupled plasma reactive-ion etching (NAURA GSE C200) with pure Ar sputtering. 200 nm thickness of lithium niobate was etched. After resist and residual cleaning, 1 μm thick silicon oxide over-cladding was deposited using plasma enhanced vapor deposition (STS PECVD).

### High-speed data transmission measurement

At the transmitter, the 56 Gbaud PAM-4 signals for all three channels were generated using an arbitrary waveform generator (Keysight M8192A, 92 Gsa/s) and amplified using linear amplifiers (SHF S807C) before sending into the modulators. The lab-made TFLN modulator features a half-wave voltage of 4.5 V and a bandwidth of 40 GHz. The commercial modulator (Fujitsu FTM7938) features a half-wave voltage of 3.5 V and a bandwidth of 25 GHz. At the receiver, after the DUT, the signals at the central channel were photo-detected using a 33 GHz receiver (PICOMETRIX BR-40D) and digitized by a real-time oscilloscope (Keysight DSOZ634A, 160 Gsa/s sampling rate, 63 GHz analog bandwidth) for offline processing. The following procedures were then performed in MATLAB for offline digital signal processing. The received signals were firstly down-sampled to 112 Gsa/s, i.e., 2 samples/symbol. A decision-directed least mean square equalizer with up to 31 taps was then used for signal equalization. Hard decision and error counting were then performed for BER measurement.

### Supplementary information

Supplementary Information for Anisotropy-free arrayed waveguide gratings on X-cut thin film lithium niobate platform of in-plane anisotropy
