# Supplementary material for: Anisotropy-free arrayed waveguide gratings on X-cut thin film lithium niobate platform of in-plane anisotropy
Source: Light Sci Appl. 2024 Jun 29;13:147. doi: 10.1038/s41377-024-01506-1 (PMC11217451; doi:10.1038/s41377-024-01506-1)
Supplement: Supplementary file 1 — Supplementary Information for Anisotropy-free arrayed waveguide gratings on X-cut thin film lithium niobate platform of in-plane anisotropy [file 41377_2024_1506_MOESM1_ESM.docx]

Supplementary Information for:

Anisotropy-free arrayed waveguide gratings on X-cut thin film lithium niobate platform of in-plane anisotropy

Junjie Yi, et al.

**Supplemental Note 1: Derivation of the angle dependent effective index of an in-plane anisotropic waveguide**

Figure S1. (a) Simulated light propagation in an on-chip anisotropic waveguide along a non-principal axis of the material. Here, an artificial uniaxial anisotropic material is considered with an anisotropy 10× higher than that of lithium niobate, in order to clearly present the splitting of the wave-normal direction *k* and the energy direction (or Poynting vector) *S*. The white solid lines denote the waveguide boundaries. (b) Relations of different physical quantities for an extraordinary-polarized plane wave in an anisotropic material. Here, the electrical field *E*, the electrical displacement *D*, *k*, and *S* are in the same plane, and the magnetic field *H* and magnetic flux density *B* are perpendicular to that plane.

Let us consider a waveguide and its transverse electrical (TE) mode in a uniaxial in-plane anisotropic material as shown in Fig. S1. The waveguide is along a direction with an angle *θ* to the Y axis of the material. Figure S1(a) also presents the light propagation in this waveguide simulated with a finite-difference time-domain algorithm. It is clear that in this anisotropic situation the wave front of the TE mode is no longer perpendicular to the waveguide direction, which means that the phase direction (wave-normal direction, *k*) and the energy direction (the waveguide direction, *S*) are different. This is similar to the case of an extraordinary-polarized plane wave in a uniform anisotropic media as shown in Fig. S1(b). For such an extraordinary wave with an angle *θ* between its *S* direction and the Y axis, its energy refractive index (defined as the ratio between the light speed in vacuum and the energy velocity, *c*/*v_s_*), or mode effective index for a waveguide *n*_eff_, can be expressed as [1]:

$n_{\mathrm{eff}}^{2}\left( \theta\right)=n_{\mathrm{eff}}^{2}\left( 0^{\circ} \right)\cos^{2} \theta+n_{\mathrm{eff}}^{2}\left( 90^{\circ} \right)\sin^{2} \theta$ (S1)

Although this equation is actually different from Eq. (3) in the main text, we will show in the following that Eq. (3) is a reasonable approximation to Eq. (S1).

Let us take a difference between the square of the left- and right-hand sides of Eq. (3) as

$$n_{\mathrm{eff}}^{2}\left( \theta\right)-\left[ n_{\mathrm{eff}}\left( 0^{\circ} \right)\cos^{2} \theta+n_{\mathrm{eff}}\left( 90^{\circ} \right)\sin^{2} \theta\right]^{2}$$

$=n_{\mathrm{eff}}^{2}\left( \theta\right)-\left[ n_{\mathrm{eff}}^{2}\left( 0^{\circ} \right)\cos^{2} \theta+n_{\mathrm{eff}}^{2}\left( 90^{\circ} \right)\sin^{2} \theta\right]+\left[ n_{\mathrm{eff}}\left( 0^{\circ} \right)-n_{\mathrm{eff}}\left( 90^{\circ} \right) \right]^{2}\cos^{2} \theta\sin^{2} \theta$ (S2)

Substituting Eq. (S1), we can rewrite the above equation as

$$n_{\mathrm{eff}}\left( \theta\right)-\left[ n_{\mathrm{eff}}\left( 0^{\circ} \right)\cos^{2} \theta+n_{\mathrm{eff}}\left( 90^{\circ} \right)\sin^{2} \theta\right]$$

$=\left\{ \left[ n_{\mathrm{eff}}\left( 0^{\circ} \right)-n_{\mathrm{eff}}\left( 90^{\circ} \right) \right]^{2}\cos^{2} \theta\sin^{2} \theta\right\}/\left\{ n_{\mathrm{eff}}\left( \theta\right)+\left[ n_{\mathrm{eff}}\left( 0^{\circ} \right)\cos^{2} \theta+n_{\mathrm{eff}}\left( 90^{\circ} \right)\sin^{2} \theta\right] \right\}$

$<{\frac{1}{4}\left[ n_{\mathrm{eff}}\left( 0^{\circ} \right)-n_{\mathrm{eff}}\left( 90^{\circ} \right) \right]}^{2}$ (S3)

The right-hand side of Eq. (S3) is close to 0 for a material with a small anisotropy. For example, for lithium niobate discussed here, the right-hand side of Eq. (S3) is in the order of 10^-4^. Actually, the fittings to the numerically simulated *n*_eff_ and *n_g_* curves using Eq. (3), as shown in Fig. 1(b) in the main text, suggest also root-mean-square errors (RMSEs) of about 4×10^-5^ and 2×10^-4^, respectively. This means that Eq. (3) in the main text is indeed an accurate estimation for the angle dependent *n*_eff_ and *n_g_* on X-cut TFLN.

**Supplemental Note 2: Comparison of different design strategies for AWGs on X-cut TFLN**

Figure S2. Fitting to the numerically simulated (a) *n*_eff_ and (b) *n_g_* using different strategies. The red curves are fittings using Eq. (3). The blue curve in (a) is the fitting using an angle independent *n*_eff_, i.e., a horizontal straight line. The blue curve in (b) is the fitting using a proportion relation to *n*_eff_.

In this section, we will analyze the induced phase errors using different design strategies for AWGs in X-cut TFLN.

The first strategy is to totally ignore the anisotropy and adopts an angle independent effective index for designing the arrayed waveguides. This is the most intuitive one, and will result in a large phase error. As shown in Fig. S2(a), the fitting of the simulated *n*_eff_ curve to a horizontal straight line would give a RMSE of 2×10^-2^. This strategy did not give a working AWG as discussed in the main text.

The second strategy is to fine-tune the length of each arrayed waveguide to fulfil the phase delay condition. This is a brutal approach, as one has to design the route and length of each waveguide separately. Additionally, if the phase delay condition, i.e., Eq. (1) in the main text, is maintained by adjusting the waveguides, one sufficient condition to fulfill also the group delay condition, i.e., Eq. (2), requires that the effective index is in proportion to the group index, i.e., $n_{g}\left( \theta\right)=a\cdot n_{\mathrm{eff}}\left( \theta\right)$, where *a* is an arbitrary coefficient. We can also perform a fitting using the above relation to the simulated *n_g_* curve as shown in Fig. S2(b), which also gives a large RMSE of 1×10^-2^. This indicates that in general cases it is unlikely for the phase and group delay conditions to be fulfilled simultaneously.

As mentioned before, using the strategy proposed in this paper, the fittings using Eq. (3) to the simulated *n*_eff_ and *n_g_* curves give RMSEs of 4×10^-5^ and 2×10^-4^, respectively. As compared to the previous two strategies, the RMSEs here are two to three orders of magnitude smaller. The RMSEs can be used to roughly estimate the length of the longest waveguide *L*_max_=*λ*·(RMSE)^-1^ that a designed AWG can have, in order not to give a significant phase error. Obviously, the present approach of RMSE in the order of 10^-5^ can support a waveguide of centimeter length. The other two approaches can only support a waveguide length of tens of microns, which is difficult to fit in with any practical AWG designs. Of course, this discussion for the AWG size is rough and likely under-estimated. Full numerical calculations are still necessary for detailed analyses as shown in the main text. Nevertheless, it is evident that the present approach can improve the performance or size of an AWG in X-cut TFLN by about two to three orders of magnitudes as compared to the other two.

To demonstrate that the proposed strategy can also be applied on AWGs with longer arrayed waveguides, we designed one device with a configuration of 1×100×6.25 GHz on x-cut TFLN [2]. The structural parameters of such an AWG are listed in Tab. S1. As shown in Fig. S3, its footprint is about 6 mm×6 mm, and good performances with crosstalk between adjacent and nonadjacent channels of -10 dB and -15 dB, respectively, can be still obtained.

Table S1. Parameters of the designed 100-channel AWG at C-band.

| **Configuration** | ***m*** | ***N*** | $\boldsymbol{w}_{\boldsymbol{a}}$ **(μm)** | $\boldsymbol{d}_{\boldsymbol{a}}$ **(μm)** | $\boldsymbol{L}_{\mathbf{FPR}}$ **(μm)** | $\boldsymbol{w}_{\boldsymbol{o}}$ **(μm)** | $\boldsymbol{d}_{\boldsymbol{o}}$ **(μm)** |
| --- | --- | --- | --- | --- | --- | --- | --- |
| 1×100×6.25 GHz | 250 | 100 | 2.9 | 3.4 | 1000 | 1.8 | 2.4 |

Figure S3. (a) Layout of an AWG with 100 output channels and 6.25 GHz channel spacing on x-cut TFLN. (b) Simulated spectral response of this design.

**Supplemental Note 3: Wavelength responses of the 8×8 AWG router**

Figure S4. Wavelength responses of all input channels to all output channels of an 8×8×200 GHz AWG wavelength router using Design-4 of Tab. 1 in the main text.

**Supplemental Note 4: Design of the 90° Euler bend**

The 90° Euler-bend is composed of a pair of 45° modified-Euler-bends, which are determined by the maximal and minimal curvature radii *R*_max_ and *R*_min_ [3]. In this case, the effective radius *R*_eff_ is defined as the radius of an equivalent circular arc bend connecting the two ends of the 90° Euler-bend, as shown in Fig. S5(a). When choosing the maximal curvature radii *R*_max_, the main consideration is the coupling loss between the optical mode in the bending waveguide and straight waveguide. In Fig. S5(b), it can be seen that as the radius decreases the field distribution of the TE0 mode in the bend deviates from that in the straight waveguide and the coupling loss increases. Therefore, taking into account the size of the device, *R*_max_ of 300 μm is chosen, which corresponds to a negligible theoretical coupling loss (<10^-4^ dB). Similarly, the principle of selecting the minimum radius *R*_min_ (50 μm) is to minimize mode leakage loss while keeping a compact device size simultaneously. Additionally, we adopt a 25-μm-long linear taper to connect the straight arrayed waveguides (2 μm in width) and 90° Euler-bend (0.7 μm in width). According to Fig. S5(c), the transmission loss of this taper is below 0.01 dB. The above optimization procedure finally gives a compact bending structure of *R*_eff_=82 μm, which exhibits a low loss for the TE0 mode and high losses for higher-order modes, as discussed in the main text.

Figure S5. (a) Schematic diagram of the 90° Euler-bend and the equivalent arc bend. (b) Transition loss between a straight waveguide and a bending waveguide with different radii *R*_max_. (c) Transmission of the linear taper connecting the straight arrayed waveguide (2 μm) and the bend (0.7 μm) with respect to the length of the taper *L.*

**References:**

[1] M. Born and E. Wolf, Principles of Optics, 7th (expanded) edition, Cambridge University Press, Cambridge, Sec. 15.2 (1999)

[2] H. Tu, Y. Zhang, G. Li, X. Dai, Y. Wu, Y. Zhang, H. Li, Q. Lu, M. Lu, W. Guo, 100-Channel Arrayed Waveguide Grating Based on Thin Film Lithium Niobate on Insulator (LNOI), J. Lightw. Technol., Early access, (2024).

[3] X. Jiang, H. Wu, D. Dai, Low-loss and low-crosstalk multimode waveguide bend on silicon, Optics Express 26, 17680-17689 (2018).
